# Online monitoring of patient self-reported adverse events in early phase clinical trials: Views from patients, clinicians, and trial staff

**DOI:** 10.1177/1740774520972125

**Published:** 2020-11-24

**Authors:** Fiona Kennedy, Leanne Shearsmith, Michael Ayres, Oana C Lindner, Lewis Marston, Alison Pass, Sarah Danson, Galina Velikova

**Affiliations:** 1 Patient Reported Outcomes Group, Section of Patient-Centred Outcomes Research, Leeds Institute of Medical Research (LIMR) at St James’s, University of Leeds, Leeds, UK; 2 Sheffield Experimental Cancer Medicine Centre, University of Sheffield & Sheffield Teaching Hospitals NHS Foundation Trust, Sheffield, UK

**Keywords:** Adverse events, symptoms, patient-reported outcome, clinical trial, Internet

## Abstract

**Background/aims:**

New classes of cancer drugs bring a range of unknown and undesirable adverse events. Adverse event monitoring is essential in phase I trials to assess toxicity and safety. In phase II, the focus is also on efficacy but robust data on adverse events continue to inform the safety and the adverse event profile. Standard, clinician-led monitoring has been shown to underestimate patients’ symptoms. Hence, patient-reported adverse event monitoring has been argued to complement and improve the information on adverse events in early phase clinical trials. With advances in information technology, real-time patient self-reported adverse events in trials are feasible. This study explored the experiences and procedures for reporting adverse events in early phase trials among patients, clinical staff, and trial staff, and their views on using an electronic patient-reported outcome adverse event system in this setting.

**Methods:**

Qualitative interviews were conducted with patients, purposively sampled across ages, gender, and different phases of trials, and with clinical and trial-related staff involved in early phase trials (e.g. consultants, research nurses, hospital-based trial assistants/data managers, trial unit management staff). Interviews explored patient experiences and views on current adverse event reporting processes and electronic patient-reported outcome adverse event reporting. Framework analysis techniques were used to analyse the data.

**Results:**

Interviewees were from two hospital trusts with early phase portfolios in England and a trial unit, and included sixteen patients, five consultants, four research nurses, five hospital-based trial staff, and two trial unit staff. Interviews identified three key themes (patient experiences, data flow, and views on electronic patient-reported outcome adverse event reporting). Stakeholders emphasised the intensity of trials for patients and the importance of extensive information provision within the uncertainty of early phase trial drugs. Regular face-to-face appointments for patients supplemented by telephone contact aimed to capture any adverse events. Delayed or under-reporting of mild- or low-severity symptoms was evident among patients. Hospital-based staff highlighted the challenges of current data collection including intense timescales, monitoring by trial sponsors, and high workload. Positive views on electronic patient-reported outcome adverse events highlighted that this could provide a more comprehensive and accurate view on the side effects of new drugs. Clinical staff emphasised patient safety and the need for clear responsibilities for monitoring. The need for careful decision-making about data flow and symptom attribution was highlighted; with trial unit staff emphasising the need for clinician review.

**Conclusion:**

Technology advances mean it is timely to explore the benefits and challenges of electronic patient-reported outcome adverse event reporting. This is a complex area warranting further consideration within the trial community. We have developed an online patient self-reporting tool and a small pilot with early phase trial patients is underway.

## Background

New treatments are continually being developed to improve outcomes for patients living with advanced cancer, which require extensive phase I–IV clinical trials before they are used in routine clinical care. Novel therapies with new modes of activity, such as targeted agents and immunotherapy, are increasingly complex and have unknown toxicity profiles that require monitoring during trials.^1,2^

In phase I trials (first-in-humans), the dose is gradually increased and safety, side effects, best dose, and timing are explored.^3^ In phase II, early evaluation of efficacy is undertaken but safety and side effects are still explored.^3^ Hereafter, we collectively refer to both these phases as ‘early phase’ and accurate reporting of adverse events is vital.^4^ Early phase trial patients can experience significant toxicity which can reduce study duration.^5^ Adverse events are traditionally clinician-recorded using the Common Terminology Criteria for Adverse Events (CTCAE),^6^ relying on the clinician’s interpretation, gauged through consultation with patients. Research has consistently demonstrated that clinicians downgrade symptom severity and under-report lower grade morbidity with implications for patients’ survival, quality of life, and trial outcomes.^7–10^

A White Paper published by Basch et al.^11^ highlighted the importance of expanding the definition of drug tolerability to give more attention to patient experience – for example, new treatments should be assessed carefully to explore overall benefit through patient-derived symptomatic adverse events data, in addition to routinely evaluated outcomes such as survival, clinician-derived endpoints including dose modifications, CTCAE, and healthcare utilisation. Therefore, the value of collecting patient-reported outcomes directly from patients, unfiltered by clinicians, has been increasingly highlighted.^2,11–13^ From a US Food and Drug Administration perspective, Kim et al.^14^ highlight that patient-reported data would not be reported directly to a drug regulator in isolation, instead it is complementary to guide clinical care^15^ and may trigger a clinical assessment. This may lead to enhanced patient outcomes^16–18^ and more informed drug prescribing and patient information.^11,19^ The National Cancer Institute has developed a patient-reported outcome version of the CTCAE (PRO-CTCAE), aiming to collect patient self-reported adverse events in clinical trials.^20^ This approach is clearly valuable, but there may be challenges in the early phase trial setting, as participants can be unwell and already significantly burdened by the trial process.^21–23^ Qualitative research exploring early phase trial patients’ experiences have focused on their information needs and decision-making around entering trials.^24,25^ Therefore, their views on self-reporting adverse events should be explored.

Recent technological advances mean real-time patient-reported data collection is increasingly feasible.^26–28^ Electronic adverse events are convenient for patients, increase data accuracy, reduce long-term costs, and provide large datasets.^15,16^ Trial patients often experience adverse events at home and in between their regular hospital visits; therefore, real-time online reporting may bridge the reporting gap and provide information not captured by the current outpatient methods. Furthermore, remote methods of monitoring are even more relevant for immuno-compromised trial patients who are especially vulnerable during the coronavirus disease 2019 (COVID-19) pandemic, facilitating the triage of patients and reducing the need for face-to-face clinical encounters where the risk–benefit ratio deem it appropriate.^29,30^ Electronic methods also enable the use of automated alerts for severe symptoms, which could facilitate the management of patient-reported adverse events in trials,^31^ but further work is needed to explore a variety of stakeholders’ views and whether this is acceptable in an early phase trial setting.

Recent early phase trials have explored patient-reported adverse events, although mostly using paper-based methods^32,33^ or waiting room tablet data collection,^15,34,35^ rather than remote, home-based online methods. There is limited evidence of electronic patient-reported outcome adverse events (ePRO-AEs) collection within early phase trials, although this is an emerging area.^36^ This study aimed to explore the experiences of reporting and monitoring adverse events among relevant stakeholders within two large National Health Service Trusts in England and their views of using ePRO methods in this setting.

## Methods

Semi-structured qualitative interviews were conducted by one of four researchers (L.M., O.L., M.A., and F.K.). A phenomenology qualitative approach was utilised, as it is particularly useful in under-researched areas, whereby each person’s unique view is considered meaningful and valid.^37^ The researchers were not known to patient participants and were independent from the clinical and trial staff. Researchers were all employed or seconded to the research group, thus shared an interest in patient-reported outcomes.

### Interview topic guide

The interview topic guides are depicted in Table 1. Patient interviews explored their experience of trial information provision, symptom/side effect reporting, and their views on using an online reporting system. Staff interviews explored the adverse event reporting process, and strengths, weaknesses, and challenges of implementing ePRO-AEs in this setting.

**Table 1.** Interview schedules for staff and patients.

Staff	Patients
***General views on reporting adverse events in early phase trials***	
• Tell me about the routine scheduled follow-up that monitors early phase trial patients? (timing, frequency, length of appointment, by whom, phase I versus phase II) • Patients’ key point of contact when enrolled on an early phase clinical trial? • How are adverse events monitored in early phase clinical trial patients? (phase I versus phase II) • trial? • How are adverse events recorded in early phase clinical trials? (phase I versus phase II) • Trial-related staff – Have you encountered concerning patient-reported trial data in the past? If so, what actions (if any) did you take in response to this? *(phase I/phase II)* • What information are patients given about reporting adverse events? How is it given (verbally/participant information sheets)? (phase I versus phase II) • trial? • What do patients do [to report and monitor adverse events?] out-of-hours? • trial? • Are there any key adverse events/symptoms that would be applicable/important to monitor across early phase trials?	**Information provision** • Did you feel you had enough information about the symptoms you may experience or which symptoms you should contact the clinical team about? • Were you told about any symptoms that could develop related to the trial treatment? • Who gave you this information? Written and/or verbal? • Did you feel prepared about what to do, when and who to contact while on trial? • Who were you told to contact if you had a problem? Out-of-hours? **Reporting side-effects/symptoms** • Have you had any problems with symptoms/side effects while on the trial? • Did you contact anyone about these? • If not, why not? Any other action, i.e. self-medicate/wait for routine appointment? • If you did, who did you contact? Why did you contact them? How did you get in contact? What happened following this contact/reporting?
***Views on electronic patient-reporting of adverse events in an early phase trials***	
• How often should patients be asked/reminded to complete these? Should there be an option for completion at any time if new adverse events are experienced? • If the information goes directly to the clinical trial office/database, if a patient reported a serious adverse event, should the patient’s clinical team/trial-related team be notified? (mild/moderate adverse events?) • Who would be the best person to be notified? • What level/frequency of information would you want to be notified about (summary on day/week/etc.; only severe adverse events or all adverse events)? • trial? • At what point would you want to be notified (in real-time, prior to a review appointment with the patient)? • trial? • How do you think electronic adverse event patient-reported data might impact on trial sponsors? • What do you envisage to be the strengths and weaknesses of a patient-reported adverse events being reported/recorded in early phase trials? • Any other challenges we should consider? • Anything else?	• Do you have internet access, do you use a computer regularly? • How would you feel about self-reporting your symptoms from home (in addition to review appointments you have with the trial/clinical team)? • How often/regularly would it be acceptable for you to routinely report your symptoms online? *(daily, weekly, monthly)* • Do you envisage any practical problems using a system like this? • If you were asked to do this, would you assume that this information is being shared with your clinical team? • Would you be happy for this information to be shared with your clinical team? • Can you think of any other advantages/benefits of using a system like this? • Can you think of any other disadvantages of using a system like this? • Anything else?

### Participants

Patient eligibility criteria included current or recent phase I or II trials’ participation, ability to understand and speak English, and having capacity to provide informed consent. Patients were purposively sampled aiming for one or two representatives across age (<60 years/>60 years), gender (female/male), and phase I or phase II. The clinical team sought this information from patient records, scoped eligibility and interest from patients, and if interested passed details to the researchers who made contact either face-to-face or via telephone.

Staff members were identified through key individuals within the hospital/university early phase trial units, then snowball sampling identified other relevant individuals (approached participants suggested other staff), including consultants, principal investigators (PIs), research nurses, hospital-based trials assistants and data managers, and trial unit staff. Staff were initially approached about the interview via email. The staff sampling aimed for a mixture of staff roles and number of years working in early phase trials.

### Procedure

Following ethical approval from National Health Service Research Ethics Committee (ref:16/NW/0659) and individual hospital approval, recruitment took place at two hospitals and a university-based clinical trial unit from December 2016 to November 2017. All participants received a written study information sheet and gave informed consent in writing or verbally (recorded). Interviews took place at a time and place preferred by the participant (hospital, telephone, or patient’s home), and some patient interviews took place alongside a family member. Interviews lasted between 12 and 70 min (average 32 min), were audio-recorded, then downloaded onto secure-access university server, transcribed, and anonymised. Transcripts were not reviewed by any participants as it was considered inappropriate to re-contact patients who were near the end of treatment options and staff who had clinical priorities. Data collection continued until data saturation was reached (i.e. no new issues emerging).

### Data analysis

Transcripts were coded independently by two researchers (F.K. and L.S.) using the framework approach.^38^ Framework analysis uses a systematic process of sifting, charting, and sorting the data into key themes using five stages: familiarisation, developing a thematic framework, indexing, charting and mapping, and interpretation. This method is suited to research that has specific questions, limited timescale, and an a priori set of issues.^39^ The thematic framework was based on the a priori interview schedule (Table 1), but additional emergent themes were identified. Data analysis took place between February and June 2019 and was initially completed separately (patient/staff) using the data analysis software NVIVO (https://www.qsrinternational.com/nvivo). Cross-cutting analysis then identified three key themes. Data analysis meetings were held throughout to ensure consistency of coding/interpretation.

## Results

Thirty-two interviews (patients – 11 telephone and 5 in person; staff – 9 telephone, 7 in person) were conducted. Due to limited resources, the number and reasons for participant decliners were not recorded. Table 2 illustrates the sample characteristics. The patients were aged 43—80 years (median 64 years), had various cancer diagnoses, and were split across phase I and II trials (N = 9/N = 7). Staff participants included consultants (N = 5), research nurses (N = 4), hospital-based trial staff (N = 5), and trial unit staff (N = 2) including a trial manager and statistician, and most had worked in this area for 5+ years (N = 14).

**Table 2.** Details of participant samples.

Patient sample	N (%)
*Age*	
Median	64 years
Range (years)	43–80 years
40–49	4 (25%)
50–59	2 (12.5%)
60–69	6 (50%)
0–79	3 (18.75%)
80+	1 (6.25%)
*Gender*	N (%)
Male	7 (43.75%)
Female	9 (56.25%)
*Phase I or phase II*	
Phase I	9 (56.25%)
Phase II	7 (43.75%)
*Cancer site*	
Bladder	2 (12.5%)
Brain (glioma)	1 (6.25%)
Breast	1 (6.25%)
Colorectal	4 (25%)
Head and neck	3 (18.75%)
Melanoma	2 (12.5%)
Mesothelioma	1 (6.25%)
Myeloma	2 (12.5%)
Staff sample	
*Professional role*	
Consultant	5 (31.25%)
Research nurse	4 (25%)
Hospital-based clinical trial assistant/data manager	5 (31.25%)
Trial unit staff	2 (12.5%)
*Length of time working in early phase trials (years)*	
1–2	1 (6.25%)
3	2 (12.5%)
5	2 (12.5%)
6	2 (12.5%)
7+	9 (56.25%)
*Involvement in phase I or phase II*	
Both	13 (81.25%)
Phase I only	1 (6.25%)
Phase II only	2 (12.5%)

**Figure 1.**
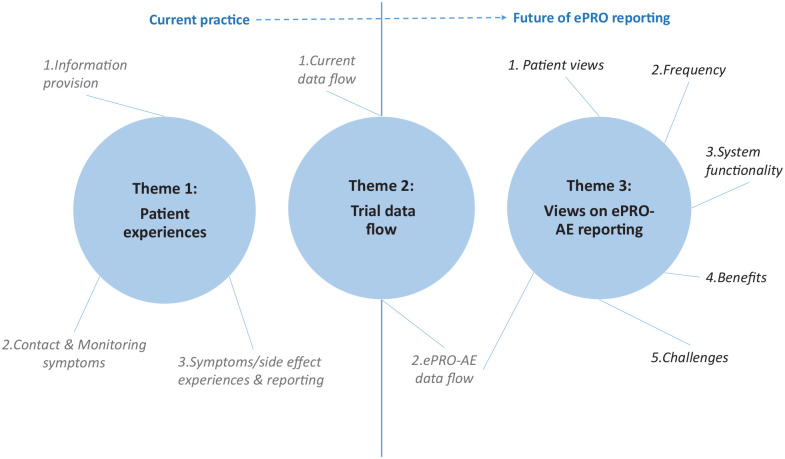
Themes and subthemes of the data analysis.

Findings are presented under three key themes that reflect the theoretical framework depicted in Figure 1. Example excerpts for each theme are provided in the supplementary tables (see Appendix 1 of Supplemental Material) and in Table 3.

**Table 3.** Theme 3: views on ePRO-AE.

Subthemes	Example excerpts
1. Patient views	1. ‘Yes I think it would be a good idea, I tend to write them in my diary…if you don’t write them down you tend to forget because it’s 3 weeks between seeing the doctor’ (Patient 12)
2. Frequency	2. ‘…If you have an open login system like that where you can just go in if you need to you could up it, couldn’t you? You could, you know, maybe once a week as a regular thing for me but if I could come in because it’s Tuesday and I’m suddenly like this I might have something I want to put in the system. If we had that flexibility as well, I don’t know’. (Patient 3) 3. ‘I suppose it does depend on the nature of the study and sort of what you might be expecting, but it’s also a balance and not wanting to make it too burdensome that it’s too frequent. So I would have thought that weekly would be sort of reasonable’. (Consultant 3)
3. System functionality	See Table 4
4. Benefits	4. ‘a patient who can report their symptoms once and twice a week may not be seen in clinic for 2 weeks. So how they felt the last week when they felt dreadful to how they feel this week, you can’t always express your symptoms the same. You know, so they might have already forgot how sick they felt and how tired they were, but at that point it were really severe. So I think it will be a really successful system to be honest’. (Clinical trial assistant 1) 5. ‘it’s more convenient for the patient, that you don’t have to try and get on the phone to somebody and get through the switchboard to the departmental secretary, to the nurse, and wait for someone to call you back. If there’s no problem you can go ahead and it’s gone in, and if there is a problem you’ve put all the info in front of, hopefully, whoever is going to come and respond’. (Patient 5) 6. ‘So it might speed up the reporting to us. It probably going to be quite a comprehensive list of things because the patients then going to think “oh I’ve got this I’ve got that”. Whereas our information currently comes from the medical records where the doctor then writes down a list of things “Mrs Smith had such and such” and he might miss 2 or 3 things off. So I think it would probably improve what we’re getting’ (Trial unit staff 2) 7. ‘we’re probably fairly good at picking up on the serious toxicities because we encourage patients to contact us, try to get them to contact us early…we may well miss sort of routinely capturing is the breadth of grade 1 and grade 2 toxicities, which actually I think sometimes patients either forget to mention or perhaps think themselves that they’re not important enough to mention…perhaps capturing the breadth of lower grade toxicities could be useful and certainly have an impact on the quality of life of the patient and their overall experience with the treatment’. (Consultant 4) 8. ‘It feels a bit like a safety net. Although there’s nobody there to talk to at the time and they’re little things that you wouldn’t ring up about, it just feels you’ve put it down in writing and somebody somewhere is going to read it’. (Patient 8)
5. Challenges	9. ‘the advantage if you like with e-reporting is that patients can do it at 10 o’clock at night or 2 in the morning, it’s a real-time capture. But there’s not necessarily going to be somebody real-time to review that. I don’t think that there’s the man power’ (Consultant 4) 10. ‘who’s going to police it, who’s going to do something about the adverse events, how quickly will it be acted on, who’s responsibility is it to look at it. It’s going to need to be accessed several times a day or ping into somebodies inbox who will action it if necessary that day because patients will assume that as they’ve reported it that that’s all they need to do’. (Consultant 2) 11. ‘I would only want to know I think grade 3 and above. So something that would, although with somethings it might be quite good to know about grade 2 you know because they’re brewing. Maybe grade 2 and above, yeah’ (Consultant 2) 12. ‘I think that it could be that it’s something that’s difficult for patients to grade their own experience perhaps. And inevitably they’re going to be grading it…compared to experiences perhaps of treatment that they’ve had before. You know, previous chemotherapy or whatever, and may use that as a benchmark. So there’s certainly, likely to be subjective interpretation of the results depending on, you know from patient to patient. And I think that the other main weakness is going to be for the patient to be able to differentiate between a treatment related side effect or symptom, compared to disease related…I think that can be quite difficult’. (Consultant 4) 13. ‘the pharma companies see everything that goes into the case notes or everything on the electronic patient record as source data. So then, I don’t know whether any of this will actually go into the patient’s record …But as soon as it does that in a trial the company will then say “well actually on ePRIME the patient said this but you only said it was a grade 1 diarrhoea…” and generate lots and lots of pharmacovigilance queries… Not saying that’s the wrong thing to do I suppose it’s that, as they interpret the regulatory framework as being you can’t go wrong with more and more information, it has its knock on consequences of somebody has to then go on and screen that and vet that. How does it marry up with what the doctors written and where’s that kind of certainty so it’s, that could be an issue’. (Consultant 1) 14. ‘If we got that big long list and therefore what do we do with it, do we assume that everything the patient’s reported needs to be reported in our trial? Whereas I think ours gets like you said a doctor filter on it and then its reported what we want…we don’t necessarily want everything that the patient happens to mention that week’. (Trial unit staff 2)

### Theme 1: patient experiences

This theme reflects the views of both patients and staff on the patient experiences on early phase trials. These trials provided patients with a last option for treatment and were described as intense with very frequent hospital visits. Clinical staff spoke about how patients were often a self-selected group: fit enough for treatment, despite being in the terminal stages of their cancer. The following patient experience subthemes explore: information provision, contact and monitoring symptoms, and symptom/side effect experiences and reporting (see quotes 1–10 – Table 5 in Appendix 1 of Supplemental Material).

#### Subtheme 1: information provision

Most patients and clinical staff were positive about the detailed information they received or gave to patients joining a trial. Specific information sheets described the study process and possible side effects, but clinical staff emphasised how they verbally discussed the inherent uncertainty owing to this being a new drug/trial. They also emphasised how new safety information may emerge, resulting in additional tests or processes. The trial unit staff emphasised the long list of potential symptoms given to patients, often split into common/expected side effects versus infrequent/serious symptoms (quote 1 – Table 5 in Appendix 1 of Supplemental Material). Some patients discussed the key symptoms they were aware of, while others described less specific information and were unclear on the symptoms they should report. Patients varied in whether they thought the information level was sufficient and reasonable or overwhelming (sometimes the case for older patients), and some candidly admitted they didn’t always read everything in detail (quotes 2/3).

#### Subtheme 2: contact and monitoring symptoms

The frequency and content of contact were largely protocol driven. Staff described an ‘intensive’ process and all participants highlighted regular (weekly, sometimes daily) appointments, especially in phase I trials, but how these might reduce as treatment is established. Patients were accepting of the intensive schedules and felt well looked after. The research nurse was usually the patients’ primary contact, and most were clear on the process/contact numbers to report symptoms during office hours and out-of-hours. However, confidence in out-of-hours service was related to whether they had used it (quotes 4/5). Patients were encouraged to phone between appointments with new symptoms, and staff sometimes made unprompted phone calls to check on patients. In the detailed clinic review appointments (quote 6), clinical staff discussed how they aimed to record what symptoms patients experienced comprehensively and accurately as per trial sponsor requirements (quote 7).

#### Subtheme 3: symptoms/side effect experiences and reporting

The patients interviewed had experienced a range of symptoms, such as rashes, lethargy, nausea, and stomach upsets, but few had required admission. Both clinical staff and patients (but not trial unit staff) highlighted that under-reporting symptoms was common, especially low-severity symptoms. Patients did not always report symptoms immediately but instead waited for routine appointments (quotes 8/9). Clinical staff suggested that under-reporting was due to patients forgetting, not perceiving mild symptoms to be important, being an ‘expected’ side effect or concern their treatment would be stopped. Clinical staff discussed the diversity of patient recording/reporting and how real-time self-reporting may encourage more accuracy (quote 10).

### Theme 2: trial data flow

This theme reflects the flow of data in trials, both in terms of current practice and views about the future of ePRO-AE.

#### Subtheme 1: current data flow

**Figure 2.**
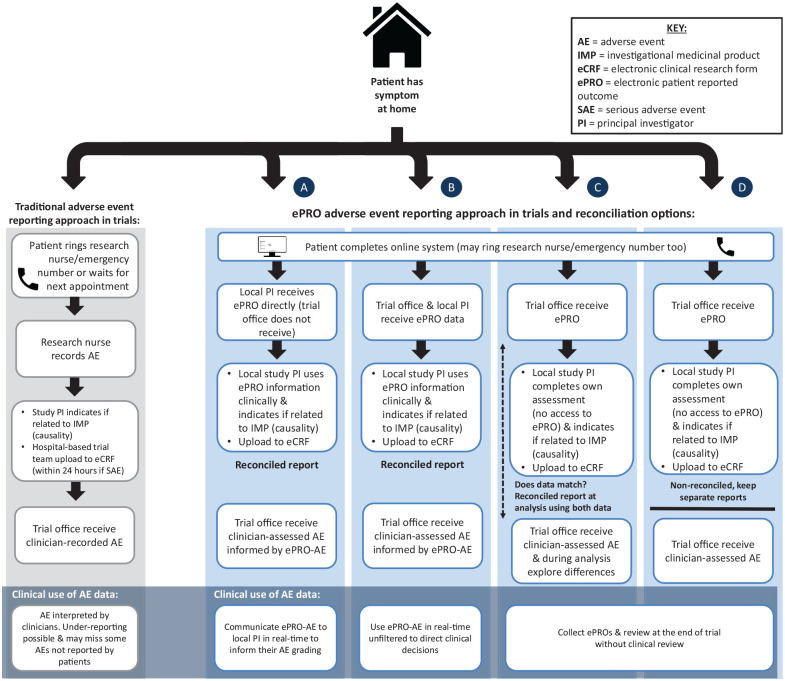
Traditional and ePRO-AE approaches. Reconciliation options informed by Di Maio et al. (2016): (a) Local PI/clinician receives ePRO direct (trial office does not receive) and uses data in their own assessment, (b) ePRO data go to both trial office and local PI to use in own assessments; (c) patient and clinician data collected separately (i.e. PI does not have access to ePRO) and only reconciled at data analysis stage (e.g. queries about discrepancies across paired data and select data that show most severe toxicities at any given time), (d) patient and clinician data collected and reported completely separately without any reconciliation.

The current well-established practice was reported (illustrated in Figure 2–‘Traditional approach’), consisting of collecting adverse events in trials through clinic visits and phone calls, recording them using CTCAE criteria by hospital-based staff who then upload to the trial sponsor while trying to determine causality (quote 1 – Table 6 in Appendix 1 of Supplemental Material).

Trial unit staff highlighted differences between trial types: phase I symptoms are collected as soon as possible in free-text format, but for phase II expected symptoms are explored per treatment cycle (quote 2). The high volume and complexity of information, along with the requirement to attribute causality and the clinical relevance of symptoms were described as particularly challenging (quote 3). Trial unit staff also highlighted how they rarely received any patient-reported data directly in early phase trials, instead all data is generally received from hospital-based teams, the only exception being a trial where patient-reported questionnaire data were received as part of the protocol outcome measures, but only explored during the final data analysis rather than in real-time (quote 4).

#### Subtheme 2: ePRO-AE data flow

Varying opinions were voiced about the potential ePRO-AE data flow (data going to clinical team versus trial office). Many patients assumed their clinical team would receive it and were happy with this, emphasising how they felt it needed to be acted on as part of routine clinical management (quote 5). Clinicians were positive about being able to cross-check the patient-reported data with their assessments, enabling a thorough monitoring of patients’ wellbeing (quote 6). However, some clinicians raised concerns about the volume of data requiring clinical review and uploading to trial sponsors, and some felt the data should go directly to the trial sponsor. In contrast, trial unit staff were resolute that ePRO-AE data should not be received by the trial office without first being reviewed by clinicians (quote 7). They viewed the online system as a tool to help clinicians establish toxicities, rather than allowing the data to directly feed into the trial outcomes.

### Theme 3: views on ePRO-AE

This theme explores participants’ views on ePRO-AE in terms of general perceptions, frequency of reporting, system functionality, benefits, and challenges. Table 3 provides example excerpt quotes illustrating the data.

#### Subtheme 1: patient views

Overall, within the patient sample, 11/16 were positive about ePRO-AE (quote 1) highlighting that it is an instant record. Two patients discussed only being able to record when feeling well and three did not have the Internet or had too few symptoms.

#### Subtheme 2: frequency

Views varied on the desired frequency for completing ePRO-AE. ‘Weekly’ was the optimal frequency among seven patients and four staff (quotes 2/3), but several also wanted flexibility to submit ad hoc reports for any new symptoms, and three other patients wanted a purely ad hoc system. Four clinical staff and a patient suggested twice weekly or daily completions, whereas five staff (both trial unit staff, two trials assistants, and a consultant) and two older patients felt monthly was sufficient.

#### Subtheme 3: system functionality

Participants made various suggestions about the functionality of any ePRO-AE system (Table 4). These included receiving reminders, question formatting, adding other or unanticipated symptoms, longitudinal graphs for clinicians, and patient prompts to contact their trial team.

**Table 4.** Theme 3: Subtheme 3: system functionality suggestions by patients and staff.

Patients	Staff
Reminders Question format – checklist+open question for other symptoms/issues User-friendly and functional Quick report function (e.g. to report no symptoms or review last submission – verify still same) Follow-up if serious or advice online Acknowledgement report received	*Staff functionality* Batching emails/alerts (not multiple emails) Flexibility to tailor to trial Function to see trends overtime (e.g. longitudinal graphs) *Patient functionality* Ad hoc access Function to add ‘other’ or unanticipated symptoms Advice to contact trial team/not substitute for phoning for advice if unwell/onus on patient to contact hospital User-friendly for patients and staff (e.g. visually inclusive – large buttons) Language simple/patient-friendly Security Access from mobiles

#### Subtheme 4: benefits

Most participants recognised the value of capturing more symptom information. Some felt that 24/7 access was particularly beneficial, and most emphasised an online system could result in less forgetting and more accuracy on symptom progression or severity in real-time (quote 4, Table 3). Participants felt that this would supplement the regular trial-related visits, guiding symptom discussions (quote 5), and some clinical staff emphasised that this could save time and reduce workload if they had access to reports before routine visits. Trial unit staff emphasised that it provided a more consistent and comprehensive hospital-based adverse event assessment, providing clinicians with more information to assess and if relevant pass onto the trial unit (quote 6).

Some clinicians felt that the system would more frequently capture low-grade toxicity, which may improve outcomes if mild symptoms are identified and managed earlier (quote 7). This mirrored patients’ views on how they did not always report mild symptoms but they valued being able to record them on the online system (quote 8).

#### Subtheme 5: challenges

Some patients and staff mentioned IT security issues such as data confidentiality, and the importance of a user-friendly system. Access to the Internet and computer skills were also raised. Patient burden was highlighted by several staff, although most patients indicated their willingness to take part in ePRO-AE alongside routine trial-related visits.

Clinical staff worried predominantly about patient safety, this being their primary responsibility (quotes 9/10), which was linked to the need to monitor ePRO-AE data. Some staff emphasised patients should continue using the current emergency/out-of-hours telephone numbers to report serious symptoms, rather than use an ePRO-AE system as they were unsure they could monitor and respond in real-time, especially with the complexity of out-of-hours staffing. However, other consultants and research nurses were keen to be alerted to severe or high-grade symptoms (grade 2+/grade 3+; quote 11) or symptoms that had changed using trial-specific algorithms. Clear staff responsibilities and a robust mechanism for receiving alerts (if used) or reviewing data were considered fundamental to ensure that serious symptoms were not missed.

One patient, two consultants, a clinical trial assistant, and both trial unit staff members highlighted the difficulty of attributing symptoms to trial drugs. Some felt that ePRO-AE could increase the ‘noise’ of patients reporting cancer-related symptoms, late effects from previous treatments, or other comorbidities (quote 12). This links to two other issues – first the potential increased workload from ePRO-AEs requiring review (quote 13) and data flow in terms of who receives the data with what responsibility (quote 14 and relates to Theme 2).

## Discussion

This study explored the experiences and views of oncology patients, clinical, and trial-specific staff on using ePRO-AE in early phase trials. The patients disclosed under-reporting of mild symptoms and suggestions that an ePRO-AE system may gather more accurate data about the breadth of toxicities experienced. Although a patient-reported symptom may not be due to the trial drug, which could be viewed as a pitfall of an ePRO-AE system, this work highlights that the current practice and possible under-reporting does not allow all potential associations to be explored. Overall, most interviewees who had experience of early phase trials as a patient or clinician viewed positive benefits and added value of an ePRO-AE system and offered useful suggestions on potential challenges and implementation issues.

Clearly our results showing under-reporting of milder symptoms is significant. From a trial-related perspective, this means the adverse event profile of new drugs is likely to miss some low-grade toxicities which could have a significant impact on patient’s quality of life and adherence to treatment.^40^ Furthermore, clinical care may be suboptimal if appropriate advice and/or supportive treatments are not being provided. Importantly, we are not aware of other studies highlighting under-reporting from the *perspective of patients*. Previous research has emphasised under-reporting among clinicians scoring adverse events,^2,7,41^ but our study suggests that filtering occurs in what patients tell their clinicians, even in the early phase trial setting.

To date, patient self-reporting of adverse events in early phase trials has been explored in a handful of studies, where patients completed adverse event monitoring on paper^32,33^ or tablet computers in the hospital.^15,34,35^ Our study highlights that most patients are willing to regularly and remotely access an online system in addition to their trial-related appointments. However, a feasibility study is now required to explore the real-time challenges/logistics, including patient burden, which may become visible once the ePRO-AE system is implemented in clinical practice.

In our study, concerns about patient safety, workload for clinical and trial staff, and data flow and monitoring of ePRO-AEs were raised by most clinical, hospital-based trial staff, and trial unit staff. Safety concerns focused on ensuring that no important symptoms are missed – either by restricting the system to non-severe symptoms or ensuring a robust mechanism for prompt clinical review of all patient-reported data. The latter could be greatly facilitated by real-time alerts based on pre-defined algorithms to multiple stakeholders. Workload concerns were raised by some clinical staff, but others felt having access to ePRO-AE data could improve their efficiency by promptly identifying and addressing ePRO-AE reported issues rather than having to collect additional data themselves. Clearly these practical implications for staff require careful exploration within any feasibility study, and they would depend on how any ePRO-AE data were used both clinically and in the trial outcomes. In the literature, various options for the reconciliation of clinician versus patient reports in trials have been suggested^2^ (see Figure 2 for ePRO approach). Clinically, the data could be shared with clinicians (options (a)/(b) in Figure 2) or not shared (options (c)/(d)), and clinicians could spend time filtering the ePRO-AE data or use unfiltered to inform clinical decisions directly. These options have differing responsibilities and workload implications for clinicians or trial-related staff and would need thorough consideration and testing in any future use of a trial ePRO-AE system.

Regardless of where the online self-reported information is transferred, it is important to inform patients where their data go, and if and when it will reviewed by their clinical team. This relates to Kim et al.^14^ who highlighted that patient-reported outcome data without clinical interpretation is not considered safety data and how clinical assessment is still needed to assess CTCAE grade and attribution. This is in line with the trial unit staff interviewed who felt clinicians should review any ePRO-AE data, rather than assuming that all patient-reported data are relevant to the trial outcomes. However, Basch argued that patient-reported data are different from clinician-reported data, they are complementary, and it is relevant to collect both to comprehensively capture the toxic impact of treatments.^8,19^

This study was conducted in the North of England, across two centres, which is a limitation. The sample was small, which means we cannot generalise the views expressed. However, the views around using ePRO-AE are likely to be of interest to other stakeholders working on trials nationally and internationally.

Focusing on patients currently or recently enrolled on an early phase trial at two hospitals also influenced the populations available for sampling in terms of ages, malignancies and treatments. Future similar studies, perhaps employing mixed methodology, could explore experiences and expectations of ePRO-AE systems across other sociodemographic and clinical groups. We note that patients recruited were all aged above 40 years. Exploring the views of ePRO-AEs in adolescent and younger adult patients would provide additional insights given their increased familiarity with technology and this may help improve clinical trial acceptability in this population.^42^ Furthermore, asking patients if they had any experience of self-reporting (paper or electronically) adverse events or other PROs in their trials would have been useful as patient’s previous experiences may have influenced their viewpoint on the proposed ePRO-AE.

Finally, we did not interview any pharmaceutical personnel or representatives of regulatory agencies, and the findings may have benefitted from that additional viewpoint. Further investigation is still required on whether ePRO-AE collection would be acceptable from an early phase regulatory perspective. However, ePRO methods will inevitably continue to grow in our increasingly technologized society, so it is important that more work is undertaken in this area.

Following this study, we are conducting a small feasibility pilot of an ePRO system among oncology patients registered on active phase I/II trials. The pilot is recruiting patients within 1 month of starting treatment and includes automated weekly reminders but the system is open should participants wish to report more frequently. The ePRO data are being sent directly to the trial office, but the research team is alerted to any severe symptoms (grade 3+). This allows the research team to inform the patient’s clinical team (requested by the trial teams) and explore the volume of alerts this generates to inform the future design of the ePRO system.

## Conclusion

Patient-reported outcome adverse events reporting is increasingly regarded as valuable and complementary to the long-standing clinician-reported toxicity process within clinical trials. The patient voice should be present in order to improve the capture of drug-related symptoms and side effects.^8^ With the capability to collect ePRO-AEs directly from patients and transfer in real-time to the sponsor or patient’s clinicians, it is timely to consider the benefits and challenges of this and how it can be practically implemented to enhance trial outcomes. In addition, lessons have been learned during the COVID-19 pandemic for using remote methods (including ePRO-AEs) to monitor some trial patients (e.g. 12+ weeks on trial, no safety concerns).^30^ Further national collaboration between clinicians, trial sponsors, and researchers is required to inform the future debate on this important area.

## Supplemental Material

sj-docx-1-ctj-10.1177_1740774520972125 – Supplemental material for Online monitoring of patient self-reported adverse events in early phase clinical trials: Views from patients, clinicians, and trial staff Click here for additional data file. Supplemental material, sj-docx-1-ctj-10.1177_1740774520972125 for Online monitoring of patient self-reported adverse events in early phase clinical trials: Views from patients, clinicians, and trial staff by Fiona Kennedy, Leanne Shearsmith, Michael Ayres, Oana C Lindner, Lewis Marston, Alison Pass, Sarah Danson and Galina Velikova in Clinical Trials
