# Supplementary material for: Online monitoring of patient self-reported adverse events in early phase clinical trials: Views from patients, clinicians, and trial staff
Source: Clin Trials. 2020 Nov 24;18(2):168–79. doi: 10.1177/1740774520972125 (PMC8010887; doi:10.1177/1740774520972125)
Supplement: sj-docx-1-ctj-10.1177_1740774520972125 – Supplemental material for Online monitoring of patient self-reported adverse events in early phase clinical trials: Views from patients, clinicians, and trial staff [file sj-docx-1-ctj-10.1177_1740774520972125.docx]

**Appendix 1 – Supplementary Tables**

**Table 5.** Theme 1: Patient experiences.

| **Subthemes:** | **Example excerpts:** |
| --- | --- |
| **1. Information provision** | *1. “Yes so in our patient information sheet we put usually a very long list of suspected adverse events and that’s because the drug companies won’t allow us not to, they like the patients to be very informed of what could happen to them. So we put in usually a pretty long list and we do ask them to let their doctor know if they have any side effects or symptoms from the drugs…so it’s usually again split up into that, “these are the ones that we are expecting to see, these are the ones that we don’t see that often but are probably the most serious” type of thing.” (Trial unit staff 2)*  *2. “I felt like I was given too much really. But no, I’m not the ideal person for this because I must admit I trust them and just skimmed through them thinking I hope I don’t get them” (Patient 8)*  *3. “there’s probably too much. But I understand they have to give you all the facts” (Patient 5)* |
| **2. Contact & Monitoring symptoms** | *4. “Well most of the time to be honest when I’ve had to, I’ve just emailed [trial research nurse] or rung [trial research nurse] because we’ve got telephone numbers. This is during the week so I’ve not had an issue on a weekend I haven’t really considered what we would do” (Patient 15)*  *5. “out of hours it’s an emergency number at the [Name of] hospital and it’s the sister that answers the phone because I have used it” (Patient 12)*  *6. “I have a clinic each week…it’s a chat through, I sit with the doctor and she’s got a checklist that’s clearly there and I report back on, that’s been fine and this is good. I describe things and she sits and scratches her head because I’m describing something in my body. I describe the tingling as wearing fur gloves inside me fingers. What can I say? You know, how can I describe it? It’s not a terrible tingling…it’s just there. It’s a funny inside feeling that I knew I didn’t have before. So we go through this stuff and she scratches her head and laughs at me and we get it clear.” (Patient 3)*  *7. “if there’s any adverse events then we have to collect data describing it, when the event started, what the grade of it was, how long it lasted for, what was done as a consequence of it, did that require a change in the dose of the medication and what is the likely association of that toxicity or presumed toxicity with the investigational agent.” (Consultant 4)* |
| **3. Symptoms/ side effect experiences & reporting** | *8. “I didn’t see them as severe so I could still tell them when I came on my Tuesday visits…a week wasn’t out of the way for me, I was quite happy treating it and mentioning when I got here” (Patient 2)*  *9. “I wouldn’t want to bother anybody or disturb anybody for something and then cause all this fuss on the phone and everything and then it all to go away and me not needed to have bothered them.” (Patient 11)*  *10. “in real-time it’s easier to get down how you feel at that given moment, rather than trivialise it, because a lot of people I find will tend to down-play symptoms because they don’t want to be denied the next cycle of treatment…if you were to give somebody a dose of a drug which was known to cause nausea for 3-5 days 2 days after the drug started. Often it’s 2 weeks later when you see them in clinic and they’ve forgotten it or they’ll down play it by then because it’s never quite that bad once you’re over it” (Research nurse 2)* |

**Table 6.** Theme 2: Trial data flow.

| **Subthemes:** | | **Example excerpts:** |
| --- | --- | --- |
| **1. Current data flow** | *1. “usually that comes down to sort of taking a really careful history, examining the patient and sort of clinical judgement, really. That might possibly involve doing imaging to see, you know, is the cancer progressing which would account for that symptom. So it’s not always entirely straightforward, but obviously you always record what the symptom is anyway. Obviously we’re required to make some sort of assessment of causality but that sometimes can be quite difficult.” (Consultant 3)*  *2. “for phase I we usually want to collect adverse events so we collect everything and that’s usually a free-text type form, so they write in the free-text of whatever the adverse event is and they’ll then give us some information about that. So things like the date it started, the date it ended so that we can monitor how long it lasted. They’ll give us a grading of how severe it was, so we can monitor how bad it got. Whether it’s attributable to the drug or not. Whereas our phase II’s we often…we will have a list of AE’s that we are expecting and then they say has the patient had it or not and what grade it got to…depending on the trials, yeah sometimes if its phase I we will ask for it in real-time, if it’s a phase II we will probably ask for it with each cycle of treatment.” (Trial unit staff 2)*  *3. “So what the companies want is they increasingly with their pharmacovigilance people is that anything you put down on a case note that becomes source data. So we’ve had, a patient had a runny nose and they’ll be getting back to you saying is that a grade 1, 2, 3 or 4 runny nose which I just think that’s ridiculous. So, certainly one of my bug bears is I think the companies collect so much data that it begins to be really difficult to sort out what’s important and what’s unimportant…the companies ask us to try and say is that clinically relevant or not. Whereas actually, ultimately in the end, it’s only when you’ve reviewed all the patients and you’ll actually be able to say there’s a trend there.” (Consultant 1)*  *4. “there is a phase 2 study that we’re just about to open that’s got, I think it’s in thyroid cancer, I’m sure it’s got a pain questionnaire in there, which would be patient reported…but no we wouldn’t be expected to do anything in terms of reporting on immediately with that data, it would be logged and it would be reported at final analysis. So we don’t kind of act in real-time on the data we receive. (Trial unit staff 1)* | |
| **2. ePRO-AE data flow** | *5. “there’s no point in having the trial unless you can share that information for it to be useful, so the fact that it could feed back to my clinician means that our conversations on a Friday could probably be that much easier or more pointed if you like or, yeah, guided” (Patient 3)*  *6. “I can gather information until it comes out me ears love, but it’s not me that’s uploading it is it. So from my workload I can see it enhancing my job because I’ll have a cross reference to say well actually I know that you’d reported this as severe but you didn’t say it was severe here. So it will help me to assess them better. But it’s double the information for the data managers, and it’s whether that would increase their workload that’s already top heavy.” (Research Nurse 1)*  *7. “we don’t collect adverse events, we collect adverse reactions and it’s filtering out what’s an event and what’s a reaction and would a) a patient be able to do that and b) would the clinician be able to do that without speaking to the patient. But I think it’s one step removed from us, I think it is a clinician tool.” (Trial unit staff 2)* | |
